# The Molecular Basis for *Escherichia coli* O157:H7 Phage FAHEc1 Endolysin Function and Protein Engineering to Increase Thermal Stability

**DOI:** 10.3390/v13061101

**Published:** 2021-06-09

**Authors:** Michael J. Love, David Coombes, Sarah H. Manners, Gayan S. Abeysekera, Craig Billington, Renwick C. J. Dobson

**Affiliations:** 1 Biomolecular Interaction Centre and School of Biological Sciences, University of Canterbury, Christchurch 8041, New Zealand; michael.love@pg.canterbury.ac.nz (M.J.L.); david.coombes@pg.canterbury.ac.nz (D.C.); sma345@uclive.ac.nz (S.H.M.); aagsampath@yahoo.com (G.S.A.); 2 Institute of Environmental Science and Research, Christchurch 8041, New Zealand; 3 Department of Biochemistry and Molecular Biology, University of Melbourne, Melbourne 3052, Australia

**Keywords:** endolysin, bacteriophage, peptidoglycan hydrolase, T4 lysozyme

## Abstract

Bacteriophage-encoded endolysins have been identified as antibacterial candidates. However, the development of endolysins as mainstream antibacterial agents first requires a comprehensive biochemical understanding. This study defines the atomic structure and enzymatic function of *Escherichia coli* O157:H7 phage FAHEc1 endolysin, LysF1. Bioinformatic analysis suggests this endolysin belongs to the T4 Lysozyme (T4L)-like family of proteins and contains a highly conserved catalytic triad. We then solved the structure of LysF1 with x-ray crystallography to 1.71 Å. LysF1 was confirmed to exist as a monomer in solution by sedimentation velocity experiments. The protein architecture of LysF1 is conserved between T4L and related endolysins. Comparative analysis with related endolysins shows that the spatial orientation of the catalytic triad is conserved, suggesting the catalytic mechanism of peptidoglycan degradation is the same as that of T4L. Differences in the sequence illustrate the role coevolution may have in the evolution of this fold. We also demonstrate that by mutating a single residue within the hydrophobic core, the thermal stability of LysF1 can be increased by 9.4 °C without compromising enzymatic activity. Overall, the characterization of LysF1 provides further insight into the T4L-like class of endolysins. Our study will help advance the development of related endolysins as antibacterial agents, as rational engineering will rely on understanding mutable positions within this protein fold.

## 1. Introduction

Bacteriophages (phages) are viral parasites of bacteria and thus have bactericidal properties. Phages have been investigated for the biocontrol of bacteria in health, agriculture and food contexts [1,2]. Their high bacterial specificity and amenability to genetic engineering are advantageous properties that allows for the development of phages with optimized bactericidal activity [3,4]. Global implementation of phage-based therapy has, however, been hindered by a poor understanding of phage biology and also regulatory hurdles [5,6,7]. Conversely, it has been discovered that some phage-encoded proteins alone, such as endolysins, have direct antibacterial activity [8], which provides a less complex system that will be easier to regulate.

Endolysins are enzymes, encoded by virulent phages, produced at the end of the phage lytic cycle. Endolysins specifically cleave the various bonds within peptidoglycan. Degradation of the cell wall causes bacterial lysis to occur, releasing progeny phages [9]. Exogenous application of recombinant endolysins can also cause cell lysis to occur, killing the bacteria [10]. This phenomenon has led to a growing number of investigations focused on the implementation of endolysins as antibacterial agents [11].

The application of endolysins against Gram-negative bacteria is generally hindered by the protective outer membrane that prevents access to the peptidoglycan [12]. Targeting Gram-negative bacteria using endolysins therefore relies on innovative approaches to penetrate through the outer membrane. Consequently, this technical hurdle has caused most to focus on characterizing and developing Gram-positive targeting endolysins [11,13,14], which has led to a number of endolysin-based clinical trials [15,16,17,18]. However, in recent years there have been successes using protein engineering to modify endolysins so they can penetrate through the outer membrane and subsequently cause cell lysis of Gram-negative bacteria [19,20]. Therefore, there is a need for characterization of the biophysical, cell lysis-causing and structural properties of Gram-negative targeting endolysins. These characterization studies may uncover endolysins with unique properties or may identify the best candidates for modification with outer membrane-penetrating functionality. Further, increasing the fundamental understanding of endolysins will allow for rational engineering to create novel endolysins with optimized properties for the effective killing of bacteria.

The objective of this work is to define the structural and functional properties of an uncharacterized endolysin, LysF1. The sequence of this endolysin was derived from *Escherichia coli* O157:H7 phage, FAHEc1, which was identified as a potential biocontrol agent against foodborne pathogenic strains and displays the useful attribute of high specificity for the Shigatoxigenic *E. coli* serogroup O157 [21,22]. Our characterization and comparative analysis of LysF1 provide a better understanding of the biology of FAHEc1, which may aid in its implementation as a phage-based biocontrol, and the elucidation of the sequence–structure relationship will support the modification of this class of endolysins into potent antibacterial agents.

## 2. Materials and Methods

### 2.1. Bioinformatic Analysis

The basic local alignment search tool, BLASTp (https://blast.ncbi.nlm.nih.gov/Blast.cgi, accessed 10 January 2021) was used to identify sequences of Protein Data Bank (PDB) structures with similarity to LysF1 [23]. These sequences were then used in a multisequence alignment using MAFFT (https://mafft.cbrc.jp/alignment/server/ accessed on 28 March 2021) that was rendered with Espript (http://espript.ibcp.fr accessed on 28 March 2021) [24,25]. The ProtParam tool from the ExPASy server was used to compute the biochemical parameters of the protein from the amino acid sequence (https://web.expasy.org/protparam/, accessed 10 January 2021) [26].

### 2.2. Expression and Purification of LysF1

The gene for LysF1 and mutants used in this study, cloned into the expression vector pET28(a)-TEV between NdeI and XhoI restriction sites, were purchased from Genscript Biotech Corporation (Piscataway, NJ, USA). This plasmid encoded the gene of interest with an N-terminal polyhistidine expression tag. BL21(DE3) cells were transformed with the plasmid and cultured in LB medium, supplemented with kanamycin (30 µg mL^−1^), at 37 °C with shaking at 180 rpm until cells reached the mid-exponential phase (OD_600_ ~0.6). Protein expression was induced with isopropyl β-_D_-1-thiogalactopyranoside (final concentration of 1 mM) at 37 °C for 3.5 h. Cells were pelleted at 8000× *g* for 15 min at 4 °C in a Sorvall™ Lynx 6000 Centrifuge (Thermo Fisher Scientific, Waltham, MA, USA). The pellet was resuspended in lysis buffer (20 mM sodium phosphate, 150 mM sodium chloride, 20 mM imidazole, pH 6.5) and cells were lysed for 15 min by sonication at 70%, with a 0.5 s on/off cycle using an ultrasonic processer UP200S (Hielscher, Teltow, Germany) on ice. Cell-free supernatant, containing soluble protein, was collected by centrifugation at 18,000× *g* at 4 °C for 15 min. 

LysF1 was purified by immobilized metal affinity chromatography followed by size exclusion chromatography. The cell-free supernatant was loaded onto a 5 mL HisTrap FF column (GE Healthcare, Chicago, IL, USA) equilibrated with lysis buffer, and the column was washed with lysis buffer until a steady baseline UV reading was reached. LysF1 was then eluted in the elution buffer (20 mM sodium phosphate, 150 mM sodium chloride, 500 mM imidazole, pH 6.5). Fractions containing LysF1 were identified with SDS-PAGE analysis and then pooled. Pooled protein was spin-concentrated to 2 mL with a Vivaspin 6, 10,000 MWCO PES spin concentrator (Sartorius, Gottingen, Germany). The aggregate was pelleted at 18,000× *g* at 4 °C for 15 min and the supernatant was loaded onto a Superdex 200 Increase 15/300 GL SEC column (GE Healthcare, Chicago, IL, USA) equilibrated with a size-exclusion buffer (20 mM sodium phosphate, 150 mM sodium chloride, pH 6.5). The purification of LysF1 to near homogeneity was visualized with SDS-PAGE analysis (Figure S1).

### 2.3. Turbidity Reduction Assay

The peptidoglycan degrading activity of LysF1 and the Glu88 mutants was evaluated against outer membrane-permeabilized Gram-negative bacterial cells and untreated Gram-positive *Staphylococcus aureus* ATCC 25923 cells by measuring reductions in optical density at 600 nm (OD_600_). Gram-negative strains *E. coli* TOP10, *Salmonella typhimurium* LT2 and *Klebsiella pneumoniae* ATCC 13883 were used. Permeabilization was performed using the chloroform-Tris-HCl technique [27]. Briefly, cells were grown at 37 °C to an OD_600_ of 0.6. Cells were pelleted and resuspended in 50 mM Tris-HCl (pH 7.7) saturated with chloroform. Cells were incubated for 45 min, then washed and resuspended in 10 mM sodium phosphate buffer to an OD_600_ of 1.0. Protein (30 µL) was combined with chloroform-treated cells (270 µL), and the change in OD_600_ was measured over time.

The effect of temperature on LysF1 activity was assessed by incubating protein (1 µg mL^−1^) at each temperature for 30 min. Protein samples were then cooled at room temperature and the residual activity measured against outer membrane-permeabilized *E. coli* TOP10 cells. 

The pH dependence of LysF1 activity was assessed by incubating protein with 270 µL of *E. coli* TOP10 cells resuspended in a universal buffer (50 mM potassium chloride, 10 mM monopotassium phosphate, 10 mM trisodium citrate and 10 mM boric acid) adjusted to different pH values with NaOH and HCl.

### 2.4. Differential Scanning Fluorimetry

The thermal stability and denaturation of LysF1 were determined by differential scanning fluorimetry using a Quantstudio 3 Real-Time PCR system (Thermo Fisher Scientific, Waltham, MA, USA). Samples were prepared with 5× SYPRO^®^ Orange and LysF1 and mutants at 0.8 mg mL^−1^. Samples were measured and prepared in triplicate (25 µL reaction volume) in a 96-well thick wall plate. Fluorescence was measured for 5 s in 0.2 °C increments from 4 °C to 95 °C.

### 2.5. X-ray Crystallography

Crystallization was performed by the sitting-drop vapor-diffusion method at 20 °C. Hexagonal prism-shaped crystals were produced at 20 °C in a drop which contained LysF1 concentrated to 15 mg mL^−1^ in size-exclusion buffer and mixed in a 1:1 ratio with 0.1 M sodium chloride, 1.6 M ammonium sulfate and 0.1 M sodium HEPES, pH 7.5. (G12, SG1™ Screen HT-96 (Molecular Dimensions, Suffolk, UK)) (Figure S2). The crystal was soaked in 15% cryoprotectant (50% ethylene glycol, 50% glycerol) and mother liquor before being snap-frozen in liquid nitrogen. Diffraction data were collected on the Australian Synchrotron MX2 beamline (Melbourne, Australia). The best data set was collected to a maximum resolution of 1.71 Å. The measured data set was scaled and quality assessed using AIMLESS [28]. Data collection and refinement stats are reported in Table 1.

The CCP4 suite of programs was used for molecular replacement and initial refinement and rebuilding [29]. The structure of LysF1 was solved with molecular replacement. First, a pruned and mutated template model of muramidase from *Acinetobacter baumannii* AB 5075UW prophage (PDB ID 6ET6) based on the sequence alignment with LysF1 was produced with CHAINSAW [30]. This model was then used to solve the structure of LysF1 using PHASER [31]. The native dataset of was initially calculated to be *P* 3_1_ 2 1; however, the solution only packed correctly in its enantiomorph, *P* 3_2_ 2 1. Refinement of the LysF1 structure was initially performed with REFMAC [32], and manual rebuilding of the structure was performed in COOT throughout refinement [33]. Finalization and model validation were conducted in PHENIX [34]. The figures presented here were produced with PyMOL.

### 2.6. Analytical Ultracentrifugation

Sedimentation velocity experiments were performed in a XL-I analytical ultracentrifuge (Beckman Coulter, Brea, CA, USA) with An-60 Ti rotor. Analytical ultracentrifugation was conducted with LysF1 at 0.32, 0.48 and 0.84 mg mL^−1^ in 20 mM sodium phosphate, 150 mM sodium chloride, pH 6.5. Sample (380 µL) and reference (400 µL) were loaded into 12 mm double sector quartz cells with epon centerpieces. Data were obtained at 50,000 rpm at 20 °C using radial absorbance scans at 281 nm and a step size of 0.003 cm over 100 scans. Buffer viscosity, density and an estimate of the partial specific based on the sequence were determined using SEDNTERP [35]. SEDFIT was used to fit the data to a sedimentation coefficient [c(*s*)] distribution model or mass distribution [c(*M*)] [36]. The residuals were inspected to assess the quality of the data (Figure S3)

### 2.7. Small-Angle X-ray Scattering

Small-angle x-ray scattering data (SAXS) were collected at the Australian Synchrotron (Melbourne, Australia) on the SAXS/WAXS beamline equipped with a Pilatus 1M detector (170 × 170 mm, effective pixel size 172 × 172 μm). A detector distance of 1600 mm, which produced a *q* range of 0.006 to 0.5 Å^−1^. 50 µL of LysF1 at a concentration of 10.43 mg mL^−1^, was injected onto an inline Superdex S200 increase 5/150 GL SEC column (GE Healthcare, Chicago, IL, USA), equilibrated with 20 mM sodium phosphate, 150 mM sodium chloride, pH 7.4 supplemented with 0.1% (*w*/*v*) sodium azide at a flow rate of 0.3 mL min^−1^. Scattering data were collected at one second exposure (wavelength = 1.0332 Å) over 400 frames using a 1.5 mm glass capillary at 25 °C. Coflow SAXS was used to minimize sample dilution and maximize the signal-to-noise ratio.

Analysis was performed using the ATSAS program suite [37]. PRIMUS was used for Guinier analysis and for producing the Kratky plot [38]. GNOM was used to calculate the pairwise distance distribution function *P*(r) via indirect Fourier transformation [39]. For a useful comparison of the experimental and computed scattering, a full model of LysF1 needed to be used. The full N-terminal His tag and missing side chains, not modelled in the final crystal structure due to missing electron density, were generated with Modeller using the Chimera interface [40,41,42]. CRYSOL was used to generate the theoretical scattering curve of the full model to compare with the experimental scattering [43].

### 2.8. Circular Dichroism

Circular dichroism measurements were performed with a J-815 spectrophotometer (JASCO Corporation, Tokyo, Japan). Wavelength scans between 200–240 nm were recorded on purified samples of LysF1 and mutants in 20 mM sodium phosphate buffer (pH 7.0) at a protein concentration of 0.26–0.30 mg mL^−1^ in a 1 mm path-length quartz cuvette in 0.2 nm steps.

## 3. Results and Discussion

### 3.1. Protein Sequence Analysis Suggests LysF1 Is a Glycoside Hydrolase Family 24 Member

Bioinformatic analysis of LysF1 was performed to identify the protein family of the endolysin and the potential catalytic mechanism. We first analyzed the primary sequence of LysF1 with ProtParam [26], identifying that the protein is 154 amino acids, with a theoretical isoelectric point (pI) of 8.89. BLASTp analysis with non-redundant protein sequences indicated that LysF1 belongs to the lysozyme-like glycoside hydrolase family 24 (GH24). This group contains phage endolysins, such as the well-studied T4 lysozyme (T4L), and bacterial autolysins, which cleave the β-1,4 glycosidic bond between *N*-acetylmuramic acid and *N*-acetylglucosamine in peptidoglycan [23,44]. A high degree of similarity to the subfamily GH24v (viral-type) sequence signature was observed [44], indicating that these conserved residues play a structural or functional role in LysF1. Several similar endolysin sequences (above 90% amino acid similarity) were identified from *Escherichia, Salmonella* and *Shigella* phages. This high level of similarity indicates that LysF1 will likely share common features with these predicted proteins and may provide insight into these yet uncharacterized endolysins.

**Figure 1:**
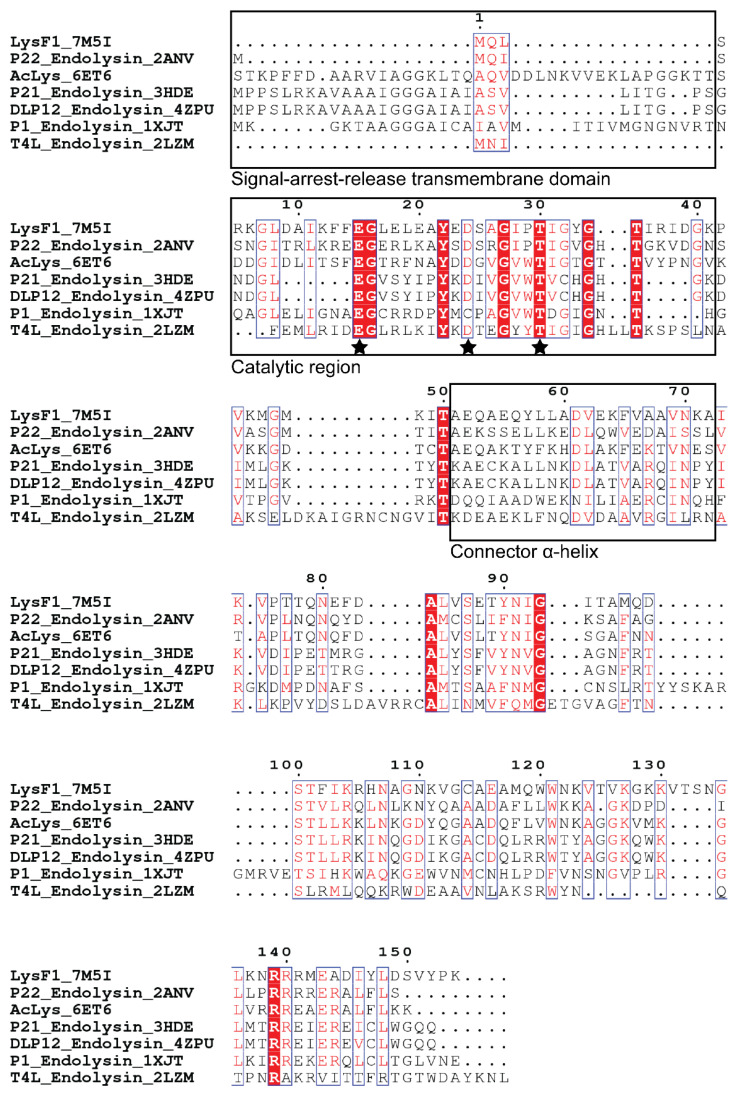
Sequence comparison of LysF1 with six closely related endolysins. Structural features of these endolysins are marked with black boxes and symbols. Putative catalytic residues are marked with a star symbol. Red boxes indicate a conserved residue. Red letters indicate similarity within a group and blue frames indicate similarity across a group.

To further assess the structural similarity that is generally conserved in this group of endolysins, we ran a protein DELTA-BLAST algorithm against those proteins with experimentally determined structures stored in the Protein Data Bank [23,45]. Six unique endolysin sequences with similarity to LysF1 were identified; *A. baumannii* phage lysozyme (43.2% identity, 95% query cover) [46], DLP12 endolysin (36.7% identity, 97% query cover) [47], P22 lysozyme mutant L86M (36.2% identity, 96% query cover) [48], P21 lysozyme (26.8% identity, 89% query cover) [49], P1 lysozyme (20.4% identity, 97% query cover) [50] and T4L (20.1% identity, 76% query cover) [51]. We performed a multisequence alignment with MAFFT to identify conserved and catalytically important residues in LysF1 (Figure 1).

Proteins that belong to the GH24v family contain a catalytic triad, consisting of a glutamate, aspartate or cysteine, and a threonine [49,52,53]. The multisequence alignment reveals that LysF1 contains these conserved residues with Glu15, Asp24 and Thr30. The conservation of these key residues suggests that LysF1 likely performs peptidoglycan degradation in a similar mechanism to T4L, the archetype of the GH24v family.

The sequence of LysF1 offers valuable information on the lytic mechanism of FAHEc1. In the canonical phage-mediated cell lysis mechanism, endolysins accumulate fully folded in the cytoplasm. Then, at the correct time, holin-mediated pore formation occurs in the plasma membrane, allowing endolysins to gain access to and hydrolyze the peptidoglycan, causing cell lysis [9,54]. An alternative lytic mechanism has recently been elucidated, where endolysins with signal peptides, signal-arrest-release regions, are transported across the plasma membrane but remain embedded in the membrane, unfolded and inactive [47,49,50]. Lysis is triggered when pin holins punch a hole in the membrane, causing the collapse of proton motive force, releasing the embedded endolysins allowing them to fold correctly and cause cell lysis [54]. Sequence comparison shows that there is no N-terminal extension on LysF1, suggesting no N-terminal signal-arrest-release region, similar to T4L and the endolysin from P22. Therefore we predict that, as LysF1 is not a signal-arrest-release endolysin, FAHEc1 lyses cells via a canonical lytic mechanism, similar to the T4 phage [55].

The sequence variation between other available structures of similar endolysins meant that we wanted to investigate our polypeptide sequence further to understand the structural implications of this diversity.

### 3.2. LysF1 Is an Endolysin That Causes Lysis of Outer Membrane-Permeabilized Gram-Negative Bacteria

**Figure 2:**
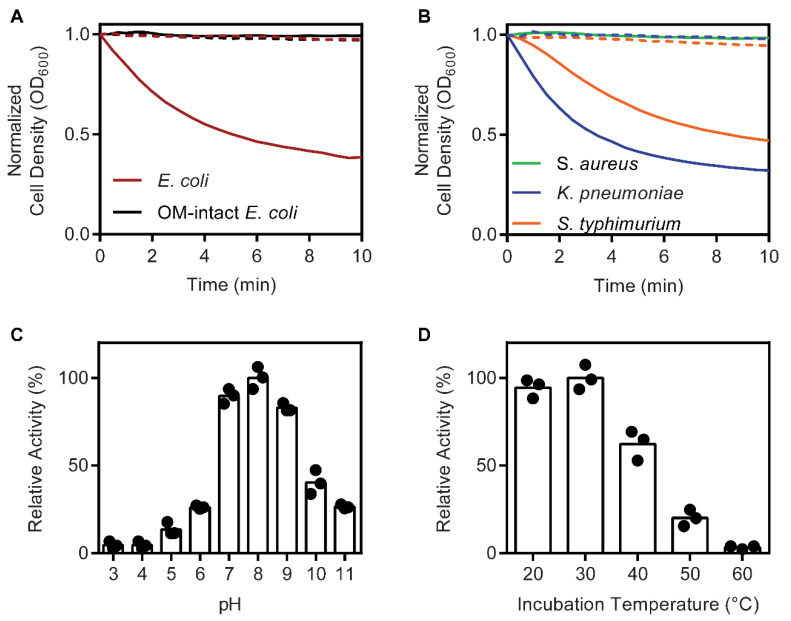
LysF1 is an active endolysin. (**A**) The incubation of LysF1 with outer membrane-permeabilized *E. coli* (red) resulted in a decrease in optical density (OD_600_) indicating lysis is occurring as a result of peptidoglycan degradation. Lysis was not observed when LysF1 was incubated with *E. coli* that had intact outer membranes (OM) (black). (**B**) LysF1 was also active against outer membrane-permeabilized *K. pneumoniae* (blue) and *S. typhimurium* (orange). LysF1 was not active against *S. aureus* (green) with no reduction in optical density measured. Dashed lines show the optical density of each cell suspension without the addition of protein. (**C**) The pH dependence of LysF1 activity measured against cells resuspended in different pH buffers. (**D**) The residual activity of LysF1 measured after 30 min incubation at each temperature. Activity is plotted relative to the highest measured activity.

Endolysin activity of LysF1 was tested by incubation with Gram-negative bacterial cells and monitoring optical density. *E. coli* cells were treated with chloroform to chemically remove the outer membrane layer and expose the peptidoglycan, which is the substrate of endolysins [14]. Addition of LysF1 to the treated *E. coli* cells produced a decrease in optical density consistent with the degradation of the peptidoglycan layer and cell lysis (Figure 2A). Addition of LysF1 to untreated cells did not reduce the optical density, demonstrating that LysF1 can only access the peptidoglycan substrate once the outer cell membrane is removed (Figure 2A). This behaviour has been reported with other Gram-negative endolysins and is a significant technical barrier to the therapeutic use of endolysins against Gram-negative bacteria [56,57,58].

To investigate the specificity of LysF1, cell lysis was measured with *S. typhimurium*, *K. pneumoniae* and *S. aureus*. Addition of LysF1 with chloroform-treated *S typhimurium* and *K. pneumoniae* produced a decrease in optical density consistent with cell lysis (Figure 2B). *E. coli*, *S. typhimurium* and *K. pneumoniae* share the same peptidoglycan chemotype (A1γ), but are more diverse at the protein level [59,60]. No cell lysis of *S. aureus* was observed (Figure 2B) despite the conservation of the targeted β(1–4) glycosidic bond [59]. This indicates that the differences in the peptidoglycan composition of *S. aureus* and the A1γ-type, such as the peptide stem or the interpeptide bridge, are important aspects of substrate recognition or degradation by LysF1. Therefore, we predict that the LysF1 endolysin is specific for the peptidoglycan substrate alone and not specific to a host species or strain. This means that LysF1 has a broader spectrum of specificity than FAHEc1, which displays near complete specificity for *E. coli* serogroup O157 [21].

Temperature and pH uniquely affect the cell lysis-causing ability of different endolysins. Therefore, we investigated these properties of LysF1. The pH dependence of LysF1 activity was measured using cells resuspended in different buffers with a defined pH. LysF1 was able to cause cell lysis across the measured pH range 3–11 with the highest activities observed between pH 7–9 (Figure 2C). Next, we investigated the impact of temperature on LysF1 activity. Protein was incubated at set temperatures for 30 min, cooled to room temperature and then the turbidity reduction activity was measured. A decrease in activity was observed after incubation at 40 °C and only 3% of activity was retained after incubation at 60 °C (Figure 2D). LysF1 displays less temperature resistance than other endolysins, such as *Salmonella* phage endolysins Lys68 [61] and Gp110 [57], which both exhibited a non-significant decrease in activity after incubation at 60 °C.

### 3.3. The Structure of LysF1

To better understand the mechanism by which LysF1 functions, we crystallized LysF1 and solved the structure to a maximum resolution of 1.71 Å. We used molecular replacement to phase the structure, exploiting the *A. baumannii* phage lysozyme, AcLys, (PDB ID 6ET6) crystal model as a template. The structure was refined to a final *R*_free_ of 20.7% and shows good geometry with no residues found in the disallowed region of the Ramachandran plot (Table 1), suggesting that our structure is of high quality.

**Figure 3:**
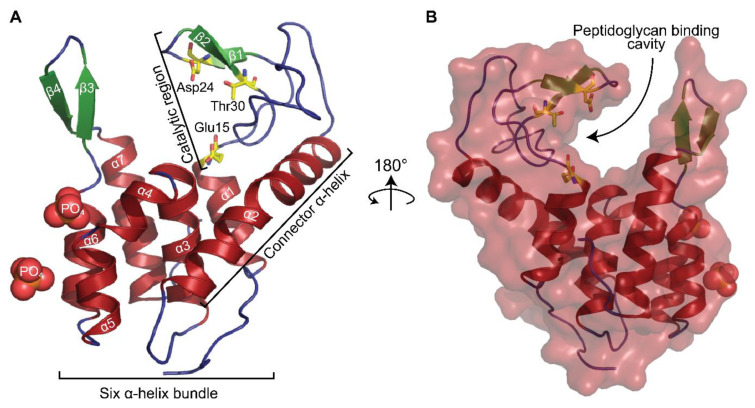
The monomeric structure of LysF1. Alpha helices in red, beta sheets in green and random coils in blue. Catalytic residues in yellow. (**A**) Cartoon representation of a monomeric structure of LysF1. (**B**) Surface representation overlaid upon the cartoon representation, showing a cavity in the catalytic region where peptidoglycan could bind.

The structure contains two protein molecules in the asymmetric unit each comprising the full endolysin sequence derived from FAHEc1 (residues 1–154) and two phosphates (Figure 3A). On chain A, only six residues of the N-terminal expression tag are also modelled while four are modelled on chain B. Residues are numbered based on the native structure (1–154). Electron density was well defined for most residues except for the side chains of Arg37, Asn39, Lys41 and Lys154 of chain A; these residues were therefore stubbed in the final model. Poor backbone density was found for Asn39–Gly40 of chain A; we predict that this could be caused by flexibility of the region. Chain A and B are similar with an average root mean square deviation (r.m.s.d.) of 0.334 (144 atoms used in the calculation). Further structure analysis is described using chain A.

The completed model reveals that the overall architecture of LysF1 consists of three regions, a catalytic loop region (Glu15–Lys41), a connector helix (α2, Ala51–Lys71) [47] and a C-terminal α-helix bundle (α3–α7, Gln79–Asp149) (Figure 3A). The protein exhibits a cleft between the catalytic loop region and the α-helix bundle where peptidoglycan is proposed to bind (Figure 3B). The catalytic loop region contains a β-hairpin (β1–β2), a structural element that is conserved across the glycoside hydrolase lysozyme super family and contains predicted catalytic residues Asp24 and Thr30 [44]. A putative catalytic residue Glu15 is positioned at the C-terminal end of α1 facing into the cleft. The catalytic loop region and C-terminal helix bundle are connected by a long α-helix, termed a ‘connector helix’, which acts as a structural backbone of the protein (Figure 3A) [47].

### 3.4. The Monomeric LysF1 Solution Structure Is Consistent with the Crystal Structure

The model determined from x-ray crystallography can occasionally misrepresent the true biological protein structure due to crystallization artefacts; in particular, the rigidity of a protein lattice can perturb protein dynamics. For example, the crystal structure contains two molecules in the asymmetric unit of LysF1 that interact, which could represent a weak biological interface or could have formed as an artefact of crystallography. Therefore, we investigated the structure of LysF1 in solution using analytical ultracentrifugation and small angle x-ray scattering.

**Figure 4:**
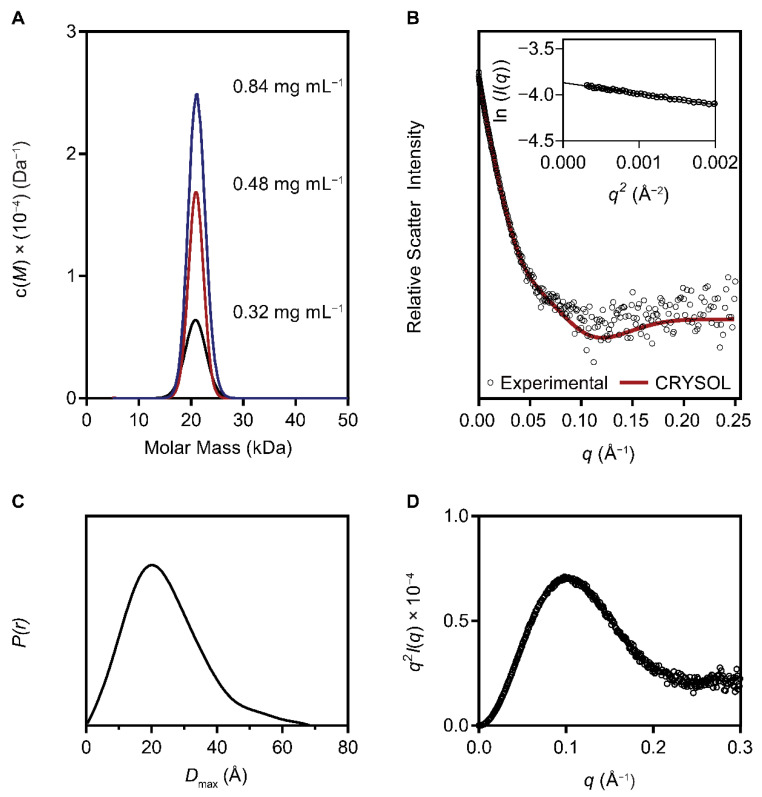
Solution structure of LysF1. (**A**) Sedimentation velocity profile of LysF1 displays a single peak at 20.2 kDa when fit to a continuous molar mass distribution model. This correlates to the molecular weight of a monomeric LysF1. (**B**) The biological relevance of the crystal structure is confirmed by a comparison of theoretical scattering of the LysF1 crystal structure to experimentally determined LysF1 having high similarity. The insert shows the linearity of the low-*q* range, indicative of high-quality data. (**C**) The pairwise distance distribution *P*(r) function determined the maximum interparticle distance was 68.39 Å. (**D**) Kratky plot displays a mostly bell-shaped profile, which shows the protein is folded. Deviation from the baseline suggests some protein flexibility.

Analysis of the structure with PDBePISA (http://pdbe.org/pisa/, accessed on 11 March 2021) [62] shows that there is only a small interface area (79.2 Å^2^) between the chains and no strong interactions exist, indicating that LysF1 is unlikely to form a biologically relevant dimer structure. However, the endolysin AcLys, which has a similar protein fold and 43.2% sequence identity, was reported to exist in a monomer–dimer equilibrium in solution [46]. Therefore, we probed the quaternary structure of LysF1 in solution using analytical ultracentrifugation. Sedimentation velocity experiments were performed at three different protein concentrations (0.32, 0.48 and 0.84 mg mL^−1^) to assess the potential equilibrium of higher order oligomeric states formation. When the data were fitted to a continuous sedimentation coefficient distribution c(*s*) and a continuous mass distribution c(*M*), a single peak is observed at 1.7 S and 20.2 kDa (Figure 4A) which is consistent with LysF1 existing as a monomer in solution. The frictional ratio (*f*/*f*_0_) of 1.37 also suggests LysF1 is asymmetric in solution. These results confirm that the two interacting LysF1 molecules within the crystal were an artefact of protein crystallization.

Next, we validated our crystal structure compared with the solution structure using SAXS. The SAXS data statistics are summarized in Table 2. A theoretical SAXS scattering was generated from the x-ray crystal structure, with the expression tag modelled using Modeller [41], and fit to the experimental scattering curves using CRYSOL (Figure 4B) [43]. The Chi-squared value (*χ*^2^) of 1.0702 indicates that the LysF1 structure is a good representative model of the structure in solution. Assessment of the experimental scattering in a low *q* region of the Guinier plot shows that the collected data were of high quality (Figure 4B, inset). The linearity of the Guinier plot signifies the homogeneity and monodispersity of the sample; nonlinearity would indicate interparticle interference or aggregation [63].

The pair-distance distribution function was determined using indirect Fourier transformation using GNOM [39]. The maximum dimension of the scattering particle (*D*_max_) was calculated to be 68.39 Å (Figure 4C). The peak is largely symmetrical between 0–40.00 Å, but exhibits a shoulder peak from 40.00–68.39 Å. This suggests that the protein in solution is mostly globular, but with some elongation [64]. The Kratky plot, calculated from the scattering, is also indicative of the shape in solution (Figure 4D). The profile of the bell-shaped curve confirms that LysF1 is folded in solution; however, the plateau above zero suggests some flexibility [65]. The molecular weight estimated from the SAXS scattering profile of 15.5 kDa is consistent with the calculated monomeric weight of LysF1.

Taken together, sedimentation velocity experiment and SAXS data confirm the validity of the LysF1 structure from the x-ray crystallography experiment and further confirm that LysF1 exists as a monomer in solution.

### 3.5. LysF1 Catalyzes Peptidoglycan Degradation in a T4L-like Mechanism

**Figure 5:**
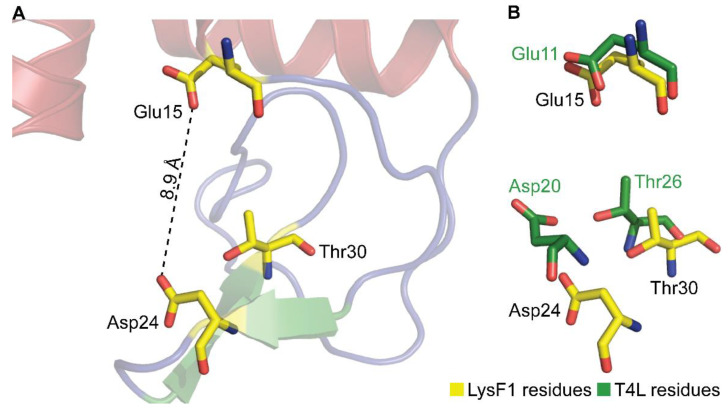
The catalytic site of LysF1. (**A**) The proposed catalytic site of LysF1 identified by sequence similarity which shows that residues Glu15 and Asp24 are positioned at an appropriate distance for the hydrolysis of the peptidoglycan. (**B**) Overlay of the catalytic residues of LysF1 (yellow) with T4L (green) shows the conservation of these key residues, suggesting LysF1 catalyzes the degradation of peptidoglycan using a similar mechanism as T4L.

Our bioinformatic analysis indicated that LysF1 belongs to the group of T4L-like endolysins that utilize a well-established mechanism involving a conserved catalytic triad [47,66]. In structurally characterized T4L-like endolysins, the catalytic domain typically contains a triad of glutamate, aspartate and threonine (Table 3). However, some variation has been observed with cysteine instead of aspartate in the P1 lysozyme [50]. Interestingly, the T4L mutant Asp20Cys also retained activity [67]. The triad is arranged in a motif of Glu(X)_8_(Asp/Cys)(X)_5_Thr, which has been observed in all published structures from this family of endolysins. LysF1 contains this conserved catalytic triad (Figure 5A), and comparison with T4L reveals that spatial orientation is also conserved (Figure 5B).

The catalytic mechanism of peptidoglycan hydrolases targeting the β-1,4 glycosidic bond between *N*-acetylmuramic acid and *N*-acetylglucosamine can be characterized by whether the anomeric center of the cleaved peptidoglycan glycoside is retained or inverted [68]. The distance between the catalytic glutamate and aspartate is a distinguishing feature of each mechanism, with an average distance of 4.3–5.9 Å observed in enzymes that retain the anomeric center and 7.2–9.5 Å typically observed when the anomeric center is inverted [47,69,70]. T4L inverts the anomeric center during catalysis [71]. The catalytic residues of LysF1 are positioned 8.9 Å apart, consistent with T4L-like endolysins, which would accommodate the peptidoglycan and water molecule between the side chains for catalysis (Figure 5A) [47]. Therefore, the catalytic site of LysF1 is consistent with a peptidoglycan hydrolase that inverts the anomeric center of the peptidoglycan glycoside during catalysis.

### 3.6. LysF1 Retains T4L-Like Architecture with Context Important Mutations

**Figure 6:**
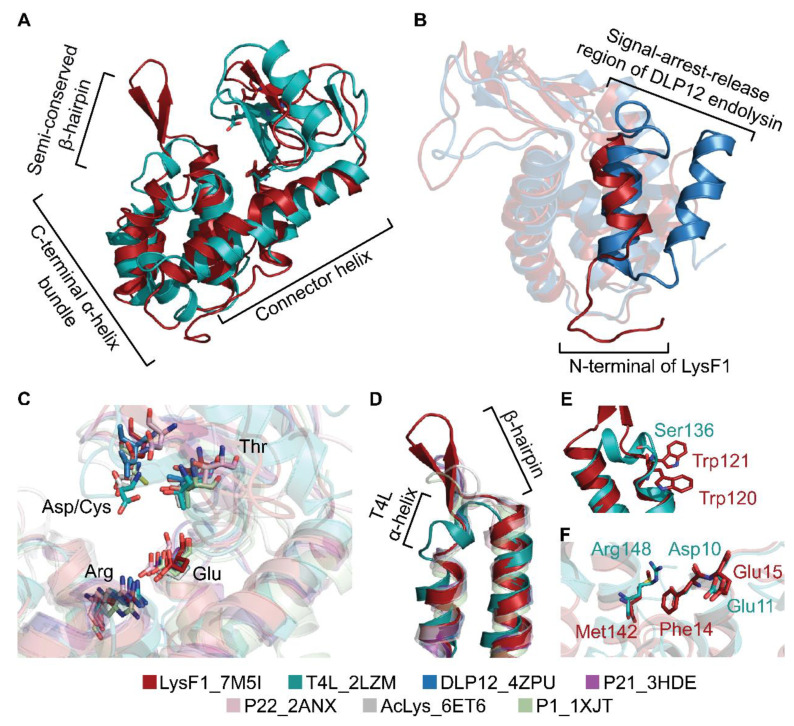
Comparison of LysF1 with related T4L-like endolysin structures. (**A**) An overlay of the cartoon model of LysF1 (red) with T4L (teal), as a representative of the T4L-like endolysin family, illustrates the conservation of the protein fold. (**B**) Overlay of the N-terminals of LysF1 with signal–arrest–release endolysin DLP12 (blue) shows that LysF1 does not contain the α-helix characteristic of signal–arrest–release endolysins. (**C**) A comparison of the active site residues highlights the conservation of Glu, Thr and Asp/Cys in terms of sequence and spatial orientation. High conservation of Arg, which is proposed to position the Glu through the formation of a salt bridge, indicates its importance. (**D**) The C-terminal bundle of this protein fold contains two α-helices that are connected via a β-hairpin of varying sizes in all compared structures except T4L which contains an α-helix. (**E**) The equivalent residue of Ser136 in T4L is a bulky tryptophan. (**F**) The salt bridge forming Asp10 in T4L is replaced with a hydrophobic phenylalanine residue in LysF1.

We have classified LysF1 as a T4L-like endolysin based upon sequence analysis (Figure 1) and inspection of the catalytic site (Figure 5B). However, LysF1 only has 20.1% sequence identity to T4L. Therefore, we performed a comparative structural analysis to further understand the sequence–structure relationship (Figure 6).

The structure of LysF1 was superimposed with T4L as a representative of the architecture observed in this protein family (Figure 6A). Despite dissimilarity in sequence identity, the superimposition of the structures revealed that the protein architecture of LysF1 is conserved (A r.m.s.d. of 2.76 was reported by RaptorX structure alignment server [72,73]). Both structures contain two domains: an α-helix bundle domain and a catalytic domain with a connector helix extending from the base of the catalytic region to beside the bundle, connecting the two domains. This architecture is observed across all structures classified as T4L-like endolysins (Figure S4).

The earlier bioinformatic analysis identified that LysF1 does not encode an N-terminal signal-arrest-release region based upon sequence analysis (Figure 1). Consistently, comparison of the N-terminal structures of LysF1 and signal–arrest–release endolysin, DLP12, confirms that LysF1 does not contain an additional α-helix that is important for insertion into the inner membrane in signal–arrest–release endolysins (Figure 6B) [47].

Comparisons of the catalytic region shows the spatial orientation of catalytic residues is conserved across the aligned endolysin structures (Figure 6C). An arginine extends from the α-helix bundle into the catalytic region forming a salt bridge to stabilize the position of the catalytic glutamate in all structures (Figure 6C) [47].

There are key residues that appear conserved across this family of proteins. Babu et al., [47] identified that in T4L, Gly12 (Gly15, LysF1 equivalent residue), Tyr18 (Tyr22), Gly23 (Gly27), Gly30 (34), Thr59 (Thr50) and Gly107 (Gly93) are conserved in endolysins with a T4L-like fold in sequence and in spatial orientation. These residues are conserved in both LysF1 and the recently characterized AcLys [46].

While there is a general conservation of protein architecture across the group, a structural difference is observed in the presence of a β-hairpin turn in the C-terminal α-helix bundle. In LysF1, a β-hairpin turn connects α6 and α7. A β-hairpin of varying sizes is seen between equivalent helices in DLP12, P21 and P1 endolysins; T4L has a small α-helix in the same position. Conversely, this region on P22 appears to be a long loop region with no α-helix or β-strand elements (Figure 6D).

The preservation of this protein architecture (Figure 6A) despite differences in the sequences (Figure 1), and the presence of numerous mutations at most of the residue positions, indicates that the fold is robust. The tolerance of T4L, the archetype of this fold, to mutation was previously shown by Rennell et al., [74]. In their study, they introduced 13 different mutations into each amino acid position and measured an approximate activity. Interestingly, point mutations that resulted in suppression of T4L activity are found in LysF1. For example, Ser136 of T4L is positioned in the small α-helical linker region between two hydrophobic core α-helices and was generally intolerant of substitution by a larger residue such as Phe and Tyr [74]. Trp120 and Trp121 were found in the similar position of LysF1 (Figure 6E). These larger residues are accommodated by other differences in the surrounding amino acid sequence that subsequently fold into different secondary structures. LysF1 contains the β-hairpin turn instead of the α-helix present in T4L. Further, a buried salt bridge between Asp10 and Arg148 in T4L was suggested to be important for stabilizing the catalytic Glu11 [74]. Asp10 is highly conserved across T4L-like sequences [52]. In LysF1, an uncharged phenylalanine residue (Phe11) is positioned into the α-helix bundle in the equivalent position but a single Glu10Phe mutation resulted in suppression of activity in T4L [74]. These observations illustrate the importance of considering the context-dependence of each mutation. A mutation may affect the phenotype of nearby residues and mutations [75]. For example, for Phe11 in LysF1 to be accommodated, there must be surrounding mutations that have coevolved when compared with the sequence of T4L. Further comparative mutagenic studies between LysF1, T4L and related endolysins would be useful to understand the role of coevolution and the impact of context-dependent mutations on the evolutionary trajectory towards this protein fold and function.

### 3.7. Improving the Function and Stability of LysF1 Through Mutation of the Hydrophobic Core

**Figure 7:**
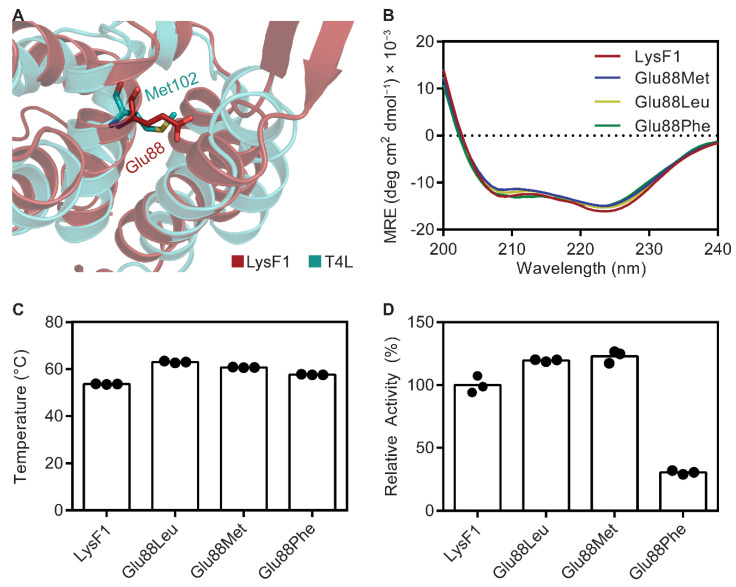
Thermal stability and relative activity of mutants of the charged core. (**A**) A magnified view of the C-terminal α-helix bundle with the crystal structures of LysF1 (red) overlaid with T4L (teal) and the residue of interest, Glu88 of LysF1, is displayed as a stick representation. The similarly positioned residue of T4L, Met102, is shown. (**B**) The circular dichroism spectra of each mutant compared with wild type LysF1. (**C**) The thermal stability of wild type LysF1 and each mutant as measured by differential scanning fluorimetry is shown. (**D**) The muralytic activity of wild type LysF1 as measured in a turbidity reduction assay of outer membrane-permeabilized *E. coli* cells compared with the three Glu88 mutants, Glu88Leu, Glu88Met and Glu88Phe. Activity is reported relative to wild type activity.

Inspection of the LysF1 crystal structure revealed the unusual appearance of a charged side chain (from Glu88) positioned within the hydrophobic core of the α-helix bundle (Figure 7A). Charged residues that are buried into hydrophobic regions are usually destabilizing for the protein fold but their presence is often found to have functional or catalytic importance [76,77]. Further, equivalent residues observed in other available structures were all hydrophobic. In T4L, Met102 occupies a similar spatial orientation as Glu88 (Figure 7A). Point mutations of Met102 were generally tolerated when replaced with hydrophobic residues, but residues with charged side changes resulted in loss of activity [74,78]. The T4L mutation Met102Lys was one of the most destabilizing single point mutations for the protein, with a decrease in thermal stability of 20.3 °C compared with the wild type [79,80]. Additionally, introduction of a glutamate at this position was reported to be inactivating [74]. We therefore hypothesized that LysF1 could have improved functionality through mutation of Glu88.

Evolutionary conservation of Glu88 amongst homologous proteins was searched for using CONSURF [81]. HMMER sequence similarity search identified 2666 unique hits, and we performed an alignment with 150 sequences. Only one other sequence was observed with a Glu in a similar sequence position. Generally, residues observed in the same positions were hydrophobic such as leucine and phenylalanine (Tables S1 and S2).

We investigated the impact of Glu88 for activity and thermal stability by overexpressing and purifying three variants of LysF1 with the point mutations: Glu88Leu, Glu88Phe and Glu88Met. Leucine and phenylalanine mutations were selected based upon their observed conservation from CONSURF. The methionine mutant was selected for its equivalent position in T4L and was previously found to be more stabilizing than a leucine [78,82], potentially because methionine can occupy the most sidechain conformations and therefore is more malleable within the structure. Initial in silico analysis with STRUM calculated that these point mutations would result in a more stable protein (ΔΔG of −0.68 for Glu88Leu, −1.03 for Glu88Phe and −0.7 for Glu88Met) [83]. Experimentally, we first determined that each mutation did not significantly affect the secondary structure using circular dichroism (Figure 7B). The introduction of a hydrophobic residue in all three variants conferred an increase of thermal stability as measured by differential scanning fluorimetry (Figure 7C). Wild type LysF1 had a melting temperature of 53.6 °C. Glu88Leu was the most stabilizing mutation, increasing the melting temperature by 9.4 °C. The methionine and phenylalanine mutations increased stability by 7.1 °C and 4.0 °C, respectively.

The impact of each mutation on activity was assessed by a turbidity reduction assay on chloroform-treated *E. coli* cells. The activity of each variant was compared with the wild type (Figure 7D). Variants Glu88Met and Glu88Leu displayed an increase in activity, 123% and 120%, respectively. However, the phenylalanine mutation reduced the activity of LysF1 to 31% of the wild type. We suggest that the reduced flexibility of the phenylalanine sidechain compared with Met, Leu and Glu could hinder the dynamics of the protein, which is likely an important property for binding and hydrolysis of the peptidoglycan.

Minor modification to the amino acid sequence of LysF1 improved the thermal stability and increased activity. LysF1 Glu88Met and Glu88Leu variants are more stable and more active than the wild type endolysin derived from FAHEc1. These findings may be useful for the genetic engineering of FAHEc1. Modifying the genome of FAHEc1 to encode one of these variants could result in a phage that is able to hydrolyze the bacterial peptidoglycan faster which may increase the rate of lysis to be more effective for the control of foodborne pathogens. Alternatively, inclusion of a phenylalanine to modulate activity of the endolysin could be an effective strategy to reduce the inflammatory response if this phage is deployed in a health context [1].

The rational engineering of endolysins, such as site-specific mutagenesis, to enhance their antibacterial activity and stability will be necessary to optimize implementation in various settings [84]. The structural characterization of many more endolysins to elucidate the sequence–structure relationship and identification of key residues will be important to support this. For example, in this study we have identified that a phenylalanine positioned in the hydrophobic core of this protein fold is deactivating and less stable compared with leucine or methionine variants. Phenylalanine is observed in similar sequences and proteins to LysF1, including AcLys [46]. AcLys displayed intrinsic moderate antibacterial activity against Gram-negative bacteria and therefore is a useful foundation for developing a novel antibacterial agent; however, AcLys contains a phenylalanine buried within its core which, based on our elucidated understanding, could be mutated to leucine or methionine to create a more stable and more active enzyme.

## 4. Conclusions

Bacteriophages and encoded endolysins are promising antibacterial candidates. Here, we report the characterization of the endolysin encoded by FAHEc1, LysF1. We confirm that LysF1 degrades peptidoglycan and causes cell lysis of chloroform-treated Gram-negative bacterial cells. Comparative sequence and structural (3D structure determined by protein x-ray crystallography) analysis reveals that despite varied sequence similarities there is a conservation of the protein architecture, illustrating the robustness of the protein fold. Further structural investigation demonstrated an unusually positioned and non-conserved glutamate within the hydrophobic core. Mutating this residue to hydrophobic amino acids resulted in a more stable, more active protein potentially elucidating an engineering pathway for optimizing similar proteins. Our characterization of LysF1 provides insight into the lytic mechanism of FAHEc1, increasing the structure–function relationship understanding of this class of endolysin, which could be useful in the future for rational engineering and the development of effective endolysin-based antibacterial agents.

## Figures and Tables

**Table 1:** X-ray data collection and refinement statistics of LysF1. Statistical values of the highest resolution shell are given in parentheses.

**Data Collection Statistics**	
Wavelength (Å)	0.953719
Temperature (K)	100
Detector	EIGER X 16M (Dectris)
Space group	*P* 3_2_ 2 1
Unit cell parameters (Å)	*a* = 67.87, *b* = 67.87, *c* = 134.44
Resolution range (Å)	44.81–1.71 (1.74–1.71)
Observed reflections	329,379 (15,760)
Unique reflections	39,743 (2042)
Mean I/σ(I)	13.1 (1.2)
CC_1/2_	0.999 (0.531)
Completeness (%)	99.8 (96.1)
*R*_merge_	0.074 (1.645)
*R*_r.i.m._	0.079 (1.761)
*R*_p.i.m._	0.028 (0.619)
Wilson B value (Å^2^)	29.9
V_M_ (Å^3^ Da^−1^)	2.30
Molecules per asymmetric unit	2
Solvent content (%)	46
**Structure and Refinement Statistics**	
*R*_factor_ (%)	18.4
*R*_free_ (%)	20.7
Number of atoms	
Macromolecules	2483
Water	299
Ligands	20
Root-mean-square deviation (r.m.s.d.)	
Bonds (Å)	0.01
Angles (°)	1.8
Average B factors (Å^2^)	
Macromolecules	34.8
Water	43.6
Ligands	59.7
Ramachandran plot, residues (%)	
Favored region	99
Allowed region	1
Disallowed region	0
PDB ID	7M5I

**Table 2:** Data analysis from the LysF1 SAXS experiment.

SAXS Data Analysis	
*I*(0) (cm^−1^)	0.021
*R*_g_ (Å)	18.53
*P(r)* analysis	
*I*(0) (cm^−1^)	0.02
*R*_g_ (Å)	18.95
*D*_max_ (Å)	68.39
Porod volume (Å^−3^)	27,014.90
Molar mass from Porod Volume (Da)	15,475
Calculated monomeric from sequence ^a^ (Da)	19,575.25

^a^ https://web.expasy.org/protparam/. Accessed 10 January 2021.

**Table 3:** Structurally characterized T4L-like endolysins.

Endolysin	Putative Key Catalytic Residues			PDB ID	Reference
LysF1	Glu15	Asp24	Thr30	7M5I	This work
T4L	Glu11	Asp20	Thr26	2LZM	[51]
P1 endolysin	Glu42	Cys51	Thr57	1XJT	[50]
P22 endolysin	Glu16	Asp25	Thr31	2ANX	[48]
P21 endolysin	Glu35	Asp44	Thr50	3HDE	[49]
DLP12 endolysin	Glu35	Asp44	Thr50	4ZPU	[47]
AcLys	Glu64	Asp73	Thr79	6ET6	[46]

## Data Availability

The x-ray crystal structure data in this study is available in the Protein Data Bank (PDB) under accession code 7M5I.

## Supplementary Materials

The following are available online at https://www.mdpi.com/article/10.3390/v13061101/s1, Figure S1: Purification gel of LysF1; Figure S2: Hexagonal prism morphology of LysF1 protein crystals; Figure S3: Residuals of c(*M*) fit indicate randomly distributed noise; Figure S4: The conserved protein architecture of LysF1. Table S1: Residue conservation for each position of LysF1; Table S2: Sequences used by CONSURF in multisequence alignment.
