# Supplementary material for: The Molecular Basis for Escherichia coli O157:H7 Phage FAHEc1 Endolysin Function and Protein Engineering to Increase Thermal Stability
Source: Viruses. 2021 Jun 9;13(6):1101. doi: 10.3390/v13061101 (PMC8228626; doi:10.3390/v13061101)
Supplement: Supplementary file 1 [file viruses-13-01101-s001.zip › MDPI Viruses Supplementary Information_Final.pdf]

viruses

MDPI

Article

The Molecular Basis for *Escherichia coli* O157:H7 Phage FAHEc1 Endolysin Function and Protein Engineering to Increase Thermal Stability

Michael J. Love [1,2], David Coombes [1], Sarah H. Manners [1], Gayan S. Abeysekera [1], Craig Billington [2,*] and Renwick C. J. Dobson [1,3,*]

[1] Biomolecular Interaction Centre and School of Biological Sciences, University of Canterbury, Christchurch 8041, New Zealand; michael.love@pg.canterbury.ac.nz (M.J.L.); david.coombes@pg.canterbury.ac.nz (D.C.); aagsampath@yahoo.com (G.S.A.); sma345@uclive.ac.nz (S.H.M.)
[2] Institute of Environmental Science and Research, Christchurch 8041, New Zealand
[3] Department of Biochemistry and Molecular Biology, University of Melbourne, Melbourne 3052, Australia
* Correspondence: craig.billington@esr.cri.nz (C.B.); renwick.dobson@canterbury.ac.nz (R.C.J.D.); Tel.: +64-3-351-0128 (C.B.); +64-3-364-2987 (R.C.J.D.)

Supplementary Information

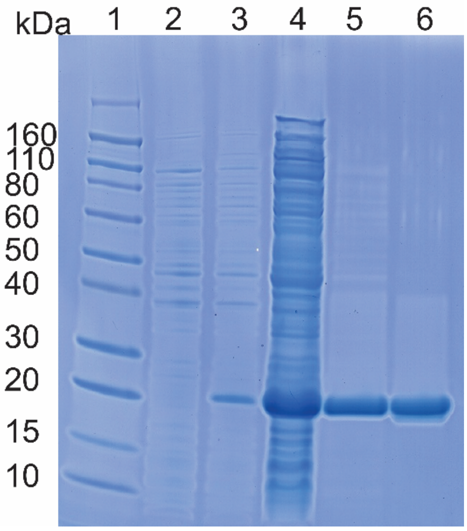

Figure S1. Purification gel of LysF1. Sodium dodecyl sulfate polyacrylamide gel electrophoresis (SDS-PAGE) analysis showing the soluble expression and increasing purity of LysF1. Lane 1, protein ladder (kDa); lane 2, uninduced expression; lane 3, whole cell lysate of induced expression; lane 4, whole cell lysate of induced expression; lane 5, pooled fraction from immobilized metal affinity chromatography; lane 6, pooled fraction from size exclusion chromatography.

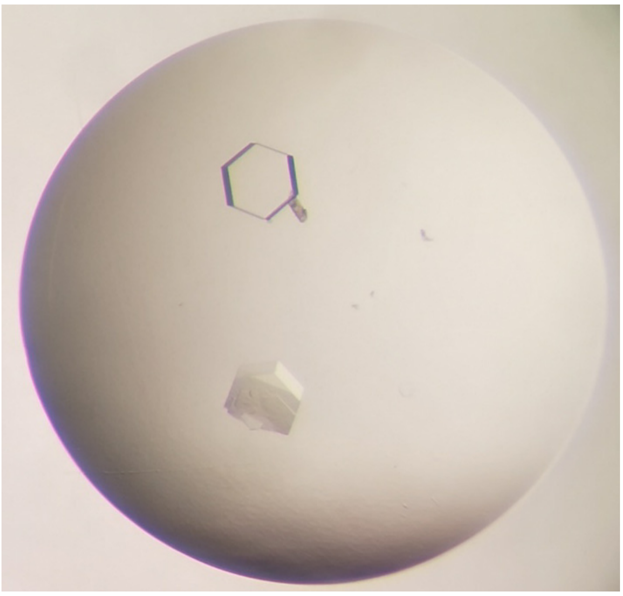

Figure S2. Hexagonal prism morphology of LysF1 protein crystals.

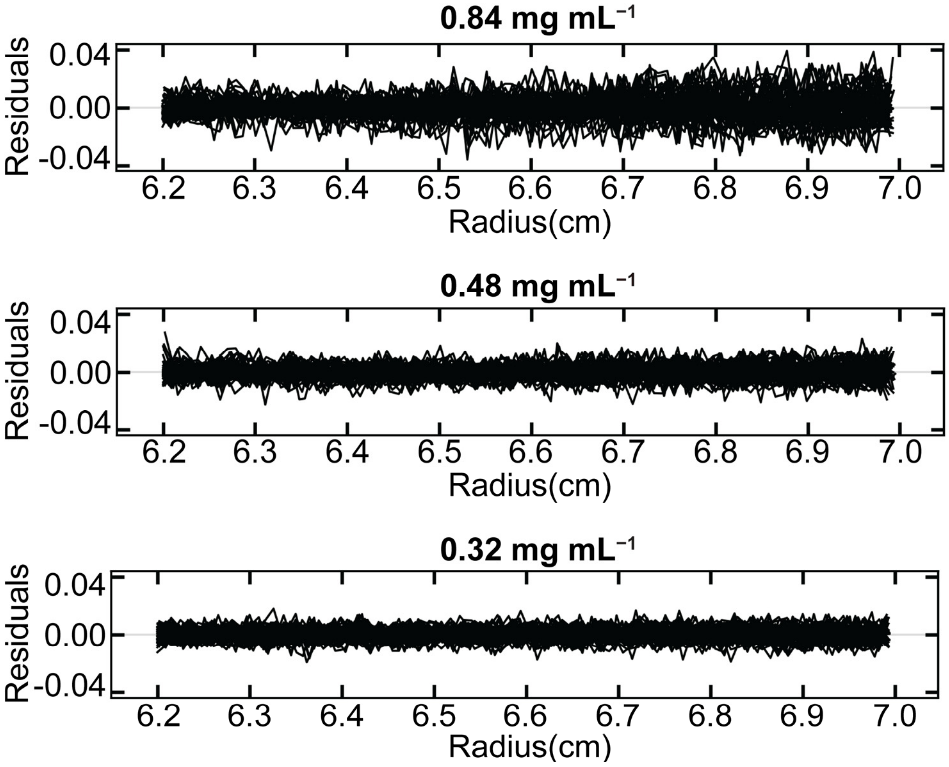

Figure S3. Residuals of c(*M*) fit indicate randomly distributed noise.

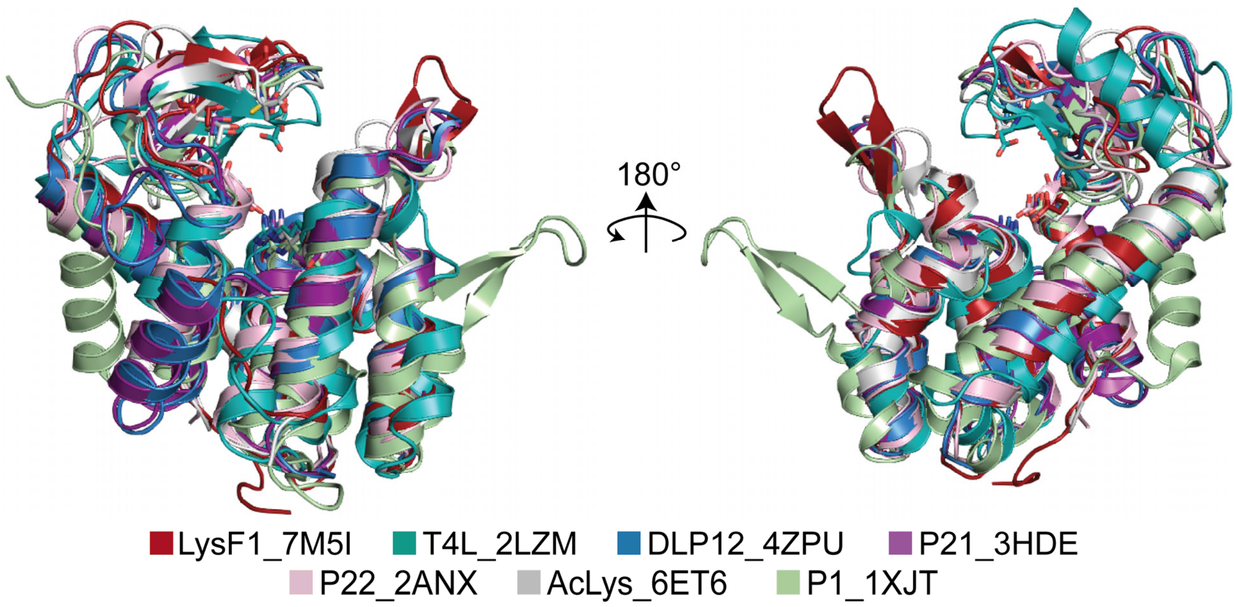

Figure S4. The conserved protein architecture of LysF1. The superimposition of the protein models of all T4L-like endolysins with available crystal structures from the protein data bank shows that the protein architecture is conserved.
